# Hiding in the dark: uncovering cancer drivers through image-guided genomics

**DOI:** 10.1186/s13059-014-0563-3

**Published:** 2014-12-20

**Authors:** Kosuke Yoshihara, Roel GW Verhaak

**Affiliations:** Department of Obstetrics and Gynecology, Niigata University Graduate School of Medical and Dental Sciences, Niigata, 951-8510 Japan; Department of Bioinformatics and Computational Biology, The University of Texas MD Anderson Cancer Center, Houston, TX 77030 USA; Department of Genome Medicine, The University of Texas MD Anderson Cancer Center, Houston, TX 77030 USA

## Abstract

Genome analysis which takes into account tumor purity leads to discovery of *PTEN* as a tumor suppressor gene in high-grade serous ovarian cancer.

See related research, http://genomebiology.com/2014/15/12/526

High-throughput approaches for molecular characterization of biospecimens enable us to comprehensively dissect cancer cells. Cohort studies of clinical tumor samples have provided valuable new insights, such as the discovery of genes that drive cancer [1]. However, surgical resection of tumor tissue often results in the inclusion of tumor-adjacent normal cells and a tumor microenvironment. As a consequence, the tissue samples under investigation in genomic and transcriptomic studies reflect heterogeneity in the number of tumor cells actually analyzed [2]. Tumor purity may be an important determinant of our ability to further uncover driver genes. In this issue of *Genome Biology*, Brenton and colleagues aimed to account for tumor purity by estimating stromal content in an unbiased fashion using *in silico* hematoxylin and eosin (H&E) slide image analysis [3].

## Defogging the ovarian carcinoma genome

The tumor microenvironment consists of immune cells, endothelial cells and fibroblasts, and is commonly referred to as tumor stroma. The stromal component contributes to the pool of DNA and RNA molecules that are used for genomic/transcriptomic analysis. While mutation analysis is generally less affected as sequencing depth can account for the relative proportion of tumor DNA, the diluting effect of non-tumor cells may be more profound on DNA copy number analysis. Genes that are homozygously deleted in the tumor cells may appear as single copy loss due to the admixture of stromal content. The same stromal component may express the gene, thus creating the impression of a hemizygously lost gene that is actively being transcribed. In order to avoid the analysis of tissue samples with very low numbers of tumor cells, tumor purity is routinely assessed. The classical and clinically widely used method is the manual evaluation of tissue sections embedded on glass slides treated with an H&E dye that visualizes individual cells and cell structures. By coloring otherwise transparent tissue sections, microscopy viewing of H&E-stained slides allows experienced pathologists and researchers to distinguish different cell types, including cancer cells, immune cells and fibroblasts. Unfortunately, this approach is affected by inter- and intra-observer biases [4]. The inclusion of tumor stroma can be avoided by using methods such as laser-capture microdissection and high-throughput cell sorting to isolate tumor cells, but these approaches are time intensive and resource consuming and for these reasons non-practical for clinical use and have only limited applicability in the research setting.

Brenton and colleagues reasoned that an automated approach may lead to higher reproducibility and accuracy of tumor purity prediction compared with manual assessment. Immunohistochemistry stains of high-grade serous ovarian cancer were matched with various molecular profiles that are available through The Cancer Genome Atlas (TCGA) [1] and comparison of the image-based stromal tissue estimates to those generated by a published and validated mRNA signature based method [2] showed a significant correlation, which suggested the accuracy of their approach. In parallel, they performed semi-automated analysis of ovarian carcinoma tissue microarray images to associate tumor-specific phosphatase and tensin homolog (PTEN) protein expression levels with clinical outcome in two independent large cohorts of ovarian cancer (Study of Epidemiology and Risk Factors in Cancer Heredity (SEARCH); Nottingham Ovarian Cancer Study (NOT)) that were part of the Ovarian Tumor Tissue Analysis consortium study [5]. The tissue microarrays provided sufficient detail to allow scoring of PTEN expression in tumor cells relative to that in stromal cells. As a result, low absolute levels of tumor-specific PTEN expression were observed in 77% of the SEARCH cohort and 52% of the NOT cohort. Moreover, the authors showed that reduced PTEN expression was significantly and independently associated with clinical outcome as well as *PTEN* copy number status in ovarian cancer. In summary, these findings suggested that *PTEN* is a putative tumor suppressor gene in ovarian carcinoma.

The results by Brenton and colleagues highlight the value of integrating H&E slide analysis, tumor purity and genomic/transcriptomic analysis. One potential caveat of this approach may be that the tissue sections used for image analysis and genomic/transcriptomic analysis may differ, resulting in discordant results. Ideally, H&E-stained slides should be obtained from tumor areas in close proximity to tumor tissue used for genomic/transcriptomic analysis.

## Clinical significance of PI3K/AKT signaling

Deactivation of PTEN through somatic mutations and deletions is a common driver event of many cancers and results in hyperactivation of the PI3K/AKT pathway. The PI3K/AKT pathway regulates cell proliferation, survival, and energy metabolism and key members such as *PI3KCA*, *AKT1, AKT2, AKT3*, *mTORC1* and *mTORC2* provide attractive therapeutic targets. DNA copy number analysis by TCGA has suggested that focal amplifications of *PIK3CA*, *AKT1*, *AKT2* and *AKT3* are frequent events in high-grade serous ovarian carcinoma and provided earlier indications that this pathway is commonly altered in this disease. However, while functional studies confirmed the activation of the PI3K/AKT axis through genetic alterations in ovarian carcinoma, this does not ubiquitously confer sensitivity to inhibitory signals, suggesting functional redundancy of the pathway [6]. Acquired inactivation of PTEN can provide resistance to PI3Kα inhibitors [7] and may present an example of how disease critical pathways can be double wired.

The TCGA analysis revealed that high-grade serous ovarian cancer is characterized by *TP53* mutation (96%), homologous recombination deficiency (51%) and somatic or germline mutation of *BRCA1* and *BRCA2* (20%) [1]. The frequent deactivation of homologous recombination pathways led to excitement over the possible application of poly (ADP-ribose) polymerase (PARP) inhibitors for treatment of ovarian carcinomas. PARP inhibitors block DNA single-strand break repair, resulting in systematic lethality with homologous recombination deficiency and are being tested in ovarian cancer clinical trials using *BRCA1/2* status as a biomarker (see ClinicalTrials.gov: NCT00753545, NCT01874353 and NCT01844986) [8]. PTEN loss also causes defects in repair of DNA double-strand breaks by homologous recombination and may provide an alternative mechanism of sensitivity to PARP inhibitors [9]. Interestingly, combination of PARP and PI3K inhibitors showed a synergistic antitumor effect in PTEN-deficient prostate tumors [10]. A phase I study of the PI3K inhibitor BKM120 and PARP inhibitor olaparib in patients with recurrent triple-negative breast cancer and recurrent high-grade serous ovarian cancer is underway (NCT01623349) and will provide important early clinical data on whether this combination therapy may provide a therapeutic advance. The standard of care for patients with high-grade ovarian cancer has not changed in several decades except for the relatively recent addition of angiogenesis inhibitors such as bevacizumab. The establishment of novel treatment modalities aimed at molecular targets may lead to improvement of prognosis for patients in dire need of better outcomes. Tumor-specific PTEN protein expression may provide one aspect of a multibiomarker panel that may guide the selection of optimal therapy to inhibit the PI3K/AKT pathway.

## Concluding remarks

A combination of genomic and image analyses has shed light on the abrogation of PTEN which was hiding behind a shade of tumor stroma and showed that loss of PTEN function can be a driver event and prognostic factor in high-grade serous ovarian cancer. These findings encourage us to re-evaluate the detection of tumor suppressor genes after taking into consideration tumor purity.

## Abbreviations

*AKT1*: v-akt murine thymoma viral oncogene homolog 1
*AKT2*: v-akt murine thymoma viral oncogene homolog 2
*AKT3*: v-akt murine thymoma viral oncogene homolog 3
*BRCA1*: Breast cancer 1, early onset
*BRCA2*: Breast cancer 2, early onset
*mTORC1*: Mechanistic target of rapamycin, complex 1
*mTORC2*: Mechanistic target of rapamycin, complex 2
NOT: Nottingham Ovarian Cancer Study
PARP: Poly (ADP-ribose) polymerase
PI3K: Phosphatidylinositol-4,5-bisphosphate 3-kinase
PTEN: Phosphatase and tensin homolog
SEARCH: Study of Epidemiology and Risk Factors in Cancer Heredity
TCGA: The Cancer Genome Atlas
*TP53*: Tumor protein p53
